# Correlates of COVID-19 Vaccine Acceptance and Hesitancy in Rural Communities in Western Kenya

**DOI:** 10.3390/vaccines11101516

**Published:** 2023-09-23

**Authors:** Fletcher Njororai, Kogutu Caleb Nyaranga, Wilberforce Cholo, Walter Amulla, Harrison Ndetan

**Affiliations:** 1 Department of Public Health, The University of Texas at Tyler, Tyler, TX 75799, USA; 2 Department of Public Health, South Eastern Kenya University (SEKU), Kitui 90200, Kenya; 3 Department of Public Health, Masinde Muliro University of Science and Technology, Kakamega 50100, Kenya; 4 Department of Public Health, Kisii University, Kisii 40200, Kenya; 5 School of Medicine at the Health Science Center, The University of Texas at Tyler, Tyler, TX 75799, USA

**Keywords:** COVID-19, vaccine hesitancy, vaccine acceptance, Kenya, correlates, Health Belief Model

## Abstract

Vaccine hesitancy is a significant global public health concern. This study sought to determine the correlates of acceptance and hesitancy regarding COVID-19 vaccines in rural populations of selected counties in Western Kenya and assess the strategies that can be used to improve COVID-19 vaccine acceptance in Kenya. The study used a quantitative research strategy with a sample of 806 individuals in the Kisumu, Vihiga, and Kakamega counties. Descriptive statistics, correlations and regression analyses were used. Of the 806 study participants, 55% were males and 45% females. Vaccine acceptance was significantly associated with being a male (AOR: 1.46, 95% CI: 1.24–1.59, *p* < 0.031), having no formal education (AOR: 2.25, 95% CI: 1.16–4.40, *p* < 0.02), working in the private sector (AOR: 5.78, 95% CI: 3.28–10.88 *p* < 0.02), and have low income (KES 0–999 (USD 0–9.16)), (AOR: 2.35, 95% CI: 1.13–3.47, *p* < 0.02). Conclusions: The current study suggests that male gender, no formal education, working in the private sector, and low income KES 0–999 (USD 0–9.6) are significant factors influencing awareness of and possible acceptance of COVID-19 vaccination.

## 1. Background

Globally, vaccine hesitancy has gained exceptional attention, triggered by its identification as a prime concern among the 10 top global public health issues or threats of the 21st century [1]. Research on vaccine hesitancy increased four-fold in the last 10 years with an exceptional rise during COVID-19 pandemic [2,3,4]. Vaccine hesitancy (VH) is defined as a delay in acceptance or refusal to be vaccinated despite the availability of vaccines and vaccination services and involves a complex interaction of many factors, including but not limited to time, place, context, and vaccine specific factors [5]. VH is a complex and multi-faceted phenomenon which, influenced and caused by numerous factors, affects the acceptance and uptake of vaccines for various diseases, not only COVID-19 [5,6]. This phenomenon, though magnified in the technological era of social media and internet, has existed since the first vaccine was administered over 200 years ago [7]. VH is not a static or homogeneous phenomenon; it can vary within and between countries, groups, population segments, time spans, and across different vaccines depending on various factors [8]. For example, an earlier study identified over 70 factors that drive or influence VH towards influenza vaccines globally [9]. According to different sources [5,10,11], VH tends to be higher in high-income countries (HICs) compared with low- and middle-income countries (LMICs). For instance, one study [10] reported that 80% of LMIC survey respondents were willing to accept vaccines and another study [5] found an even greater proportion at 95% in South Asia. A different study reported rates of VH in HICs or regions ranged from 7 to 77.9% [2].

In Kenya, at the time of this study and in 2021, there were differences in routine vaccine coverage; for example; DPT coverage was at almost 90% the recommended WHO target [12] and that for COVID-19 was at 16.7% [13], which implies differences in behaviors and acceptance of the different vaccines. Vaccine hesitancy, gaps in knowledge and the different drivers between developed and developing countries and within countries are of critical importance in addressing context-specific needs and designing effective strategies to deal with specific immunizations. For example, the generalizability of findings on vaccine hesitancy from developed countries to African countries is unclear [6,14,15,16]. More studies are imperative as society evolves and increased global interconnectedness arises through faster transportation, increased human mobility and migration, and technological advancement, environmental changes including climate change, and One Health among others, continue to transform the platforms and frameworks for local and global health.

Researchers [15,16,17] note that there remain major knowledge gaps in this area of vaccine hesitancy across and within countries globally. Much less is known about the nature and causes of vaccine hesitancy in Africa. Some countries initially faced significant limits in the ‘access and supply side’ of vaccination as well as other barriers such as cultural and religious beliefs, while for the majority other countries, conspiracy theories, misinformation, and disinformation have transformed attitudes from hesitancy to refusal and resistance [14,18,19]. Therefore, vaccine hesitancy is likely to comprise a more complex interplay of structural, political, social-cultural, psychological, and contextual influences in Africa than in high income countries.

In May 2023, the World Health Organization declared COVID-19 is no longer a global public health emergency (PHE); however, the virus is expected to stay with us for many years to come, causing infections and recurring disease. This requires global strategies to manage its possible resurgence and long-term risks in different populations across nations and different parts of the world. With the potential for new variants of the SARS-CoV-2 virus, uptake of COVID-19 vaccines remains important for controlling the spread of COVID-19 outbreaks and any future surges as variants evolve, hence the need to address VH [20,21]. Vaccinations and immunization have an unrivalled impact on global public health, with the greatest potential for further improvements in health and life expectancy, especially given the risk of increased spread of diseases at very high rates and frequency [22,23,24,25,26,27].

Globally, Africa was the continent least-affected by the COVID-19 pandemic, contrary to earlier predictions [28,29]. With a total population of approximately 1.4 billion people, Africa had only 9,420,467 confirmed cases from March 2020 to December 2022, and 257,984 deaths reported to WHO in the same period. The African continent has recorded many fewer cases and deaths to date than anticipated relative to its population size and other factors related to the mode of transmission of the virus [30,31]. Despite low vaccina-tion rates in many countries in Africa [16,32], COVID-19 infection rates remained low, yet Africa is the second largest and the second most populous continent with 18% of the world’s population as of 2021. At the time of this study, in Kenya the COVID-19 vaccine coverage rate was 16.70% [33]. As of June 2023, according to WHO [34], Kenya has a low COVID-19 coverage compared with many other countries in Africa and globally. Many African countries on the World Bank’s fragile states list grapple with poor social services due to conflicts, wars, internal migration, corruption, poverty, and displacement among other humanitarian crises, which has significant implications for global health as well as dealing with emerging and re-emerging pandemics and other diseases [33].

Kenya, with a population of over 50 million people, is a low-middle income country in sub-Saharan Africa (SSA) with about 46% of the population living below the poverty line and 75% living in rural areas [35]. This study focuses on rural communities. Understanding and addressing VH in Kenya are critical for continued efforts to provide timely health to its population. Until recently, there has been a paucity of data on vaccine hesitancy in Kenya and the consequent implications for prevention and management of future pandemics in the country [36,37]. This could be attributed to other health priorities for the country, especially with its limited resources, and/or because routine vaccinations have been generally well received and covered. This situation could cloud the need for increased attention to address VH, especially with low infection rates of COVID-19. Previous studies have shown that vaccine acceptance, hesitancy, or refusal are varied and inconsistent in each country in sub-Saharan Africa [38]. Studies specific to countries and contexts, as well as population- and age-specific studies are needed to inform successful inoculation against COVID-19 for continued prevention and management of future outbreaks [17,38]. The purpose of this study was to assess factors related to COVID-19 vaccine acceptancy and hesitancy in the western region of Kenya using the Health Belief Model [39]. The Health Belief Model has been used to guide health promotion and disease prevention programs and to explain and predict individual changes in health behaviors. Findings from this study may be helpful in providing a springboard for individual-, community-, or context-specific strategies aimed at addressing vaccine hesitancy or refusal, thereby improving COVID-19 vaccine acceptance.

## 2. Methods

### 2.1. Research Design

A cross-sectional design applying quantitative research strategies was used. The study used the Health Belief Model (HBM) to explore correlates of vaccine acceptance and hesitancy and identify ways of improving vaccine acceptance in the three counties. The Health Belief Model distinguishes and anticipates beliefs on individual health conditions and behavior. The study focused on the key salient elements of the model (perceived susceptibility, perceived severity, perceived benefits, perceived barriers to action, cues to action, and self-efficacy) to identify correlates of vaccine acceptance and hesitancy.

### 2.2. Study Area

This study was conducted in Kakamega, Vihiga, and Kisumu Counties in the western part of Kenya. Kakamega County covers an area of approximately 3050.3 km^2^. Kakamega has a population of 1,867,579 of which 897,133 (48.0%) are males, 970,406 (51.9%) are females, and 40 (0.002%) are intersex persons [40]. The county has 433,207 households. Kakamega has one county referral hospital (Kakamega County General Teaching and Referral Hospital), 12 sub-county hospitals, 47 health centers, 123 dispensaries and 44 clinics [40]. Vihiga County borders Nandi to the east, Kisumu County to the south, Siaya County to the west, and Kakamega County to the north. The County has five administrative sub-counties, namely, Luanda, Emuhaya, Hamisi, Emuhaya, Sabatia, and Vihiga. The county is further subdivided into 11 divisions, 38 locations, and 131 sub-locations [40].

Kisumu County has a population of 1,155,574, of which women make up 50.1% and men represent 49.9%. Of the total population, 64 % is under the age of 25 years [40]. Health is delivered by various private or government institutions. The county has 1 teaching and referral hospital, 5 county referral hospitals, 14 sub-county hospitals, 74 dispensaries, and 18 health centers.

### 2.3. Target Population

The study targeted all adults 18+ years residing in Kisumu, Vihiga, and Kakamega counties in western Kenya.

### 2.4. Sample Size Determination

The sample size was established using a prior analysis to determine the effect size which was taken as 10% of the population, and 80% power with a significance level of 5%. The power analysis for a one-sample proportion test in G power calculator provided a required sample size of 779. A ten percent non-response rate was added (78 more participants); hence, the sample size used in the study was 857. This number was of sufficient size to answer the study questions and reveal any statistical differences in this study. This method helps control statistical power before a study is conducted [10].

### 2.5. Sampling Procedure

Purposive sampling was used to select the three counties. Two sub-counties were selected from each county using stratified sampling based on whether they were urban or rural. Proportionate stratified sampling was adopted to select study subjects from the six sub-counties. Simple random sampling was utilized to choose two wards in every sub-county. A household list was generated based on the administrative location headed by each chief. Systematic random sampling was then used to select households in the selected wards. A representative of the eligible study subjects or house heads in the chosen households were randomly selected to participate in the study, as indicated in Table 1 below. 

### 2.6. Survey Instrument

The instrument was developed guided by previous studies considering the COVID-19 pandemic condition in Kenya [3,4,5,10,36,41,42,43]. The instrument was conducted in English and translated into local languages (Luo and Luhya). The participants who could not understand English were issued with appropriate instruments. A pilot study was conducted with 10% of the sample size, including participants chosen from areas not considered for the actual study. This was to assess the consistency, clarity, and accuracy, and necessary adjustments were made to refine the instrument.

The instrument had four sections. The first section comprised sociodemographic characteristics (age, sex, county of residence, educational level, and monthly income), and level of individual and community compliance with COVID-19 WHO containment protocols.

Section two contained questions to ascertain acceptance and hesitancy of the COVID-19 vaccine, and whether family members and friends would receive the vaccine when available (yes, no, and unsure). Those who responded no or unsure were defined to be hesitant in this study. Further, they were asked why they were not planning to be vaccinated against COVID-19.

The third section comprised questions on predictors of COVID-19 acceptance organized in terms of the Health Belief Model with questions in each salient area: perceived susceptibility, perceived severity, perceived benefit, cues to action, and SARS-CoV-2 was designed to compel people to be vaccinated. A four-point Likert scale with the possible range of responses between “one” for “strongly disagree” and “four” for “strongly agree” was employed. The Health Belief Model has been used in previous studies to explain the predictors of vaccine acceptance [44].

Section four focused on strategies to improve COVID-19 vaccine acceptance and hesitancy. These strategies were based on making social norms more salient and favorable for vaccination and included highlighting new and emerging norms in favor of vaccination, leveraging the role of health professionals to promote vaccination, and amplifying endorsements from trusted community members. These variables were further organized as described below.

### 2.7. Dependent Variables

Vaccine acceptance—defined as the degree to which individuals accept, question, or refuse vaccination. It is one of the major determinants of vaccine uptake rate, vaccine hesitancy, and consequently vaccine distribution success.

Vaccine hesitancy—refers to the delay in acceptance or refusal of vaccination despite the availability of vaccination services. Vaccine hesitancy is complex and context-specific, varying across time, place, and vaccines. It may be influenced by factors such as complacency, convenience, confidence, and more.

### 2.8. Independent Variables

Independent variables for vaccine acceptance include demographic characteristics (age, sex, occupation/employment type, education, residence/location, income, marital status).

Further independent variables include the Health Belief Model salient areas (perceived susceptibility, perceived severity, perceived benefit, cue to action); mistrust in vaccine (belief that SARS-CoV-2 was manufactured to force the public to get vaccinated); fear of the outcome of negative vaccine effects or outcomes, religious affiliations, cultural reasons/beliefs, fear or confidence in efficacy and safety of the vaccine, and mistrust in the role of health care workers in pandemic control.

### 2.9. Data Collection Procedure

A letter introducing the study, study license, and permit were presented to the respective county commissioners who granted approval to particular sub-counties of the study. Six trained research assistants were involved in data collection for a period of two months from July to August 2021 after the second COVID-19 wave in Kenya. The study participants were asked about their willingness to participate in the study after being given information on the purpose and procedures involved in the study. The respondents were given all the relevant information about the study to be undertaken to allow for voluntary consent without coercion, pressure, or undue enticement. Those who accepted to participate signed an informed consent form. Ethical approval and permission to conduct the study were obtained from the University of Eastern Africa, Baraton Institutional Research Ethics Committee (IREC), and the National Commission for Science, Technology and Innovation (NACOSTI), respectively.

### 2.10. Data Analysis

Quantitative data were cleaned, coded, and entered in SPSS version 26 program. Data editing was performed to check for any discrepancy or errors in data entry. Data analysis was performed using various techniques that involved descriptive statistics and inferential statistics. Descriptive statistics were used on the demographic characteristics of the study population and were presented as frequencies, proportions, means and standard deviations. Logistic regression analysis was used to estimate the association (crude odd ratio) between COVID-19 acceptance and demographics (*p*-value < 0.05). Further, a multivariate logistic regression model was used to assess the association between demographic characteristics and COVID-19 vaccine acceptance. The logistic regression model was used to assess the association of COVID-19 vaccine acceptance and HBM salient areas. A bivariate Spearman correlation was used to assess factors that were correlated with COVID-19 hesitancy. *p*-value ≤ 5% is considered statistically significant at 95% CI.

## 3. Results

### 3.1. Demographic Characteristics of the Participants

Out of the 857 randomly selected participants who were interviewed, 806 (94.2%) consented and completed the questionnaire. Of these, 55% were males (Table 2). Although the sex ratio varied by age group, level of education, and occupation status, a greater proportion of males were found in nearly every age group except among those aged above 70.

Differences in the sex ratios in the 18–30; 41–50; and 50–60 age groups were less prominent, with the respective ratios being 1.04, 1.08 and 1.05. They were more prominent among the participants aged 31–40 and 70 and above. Participants who had completed university had the highest male to female sex ratio (1.8); however, greater proportions of females were found among women who did not attain any formal education (53.7%). Kakamega County had the highest proportion of males compared with females (60.4% vs. 39.6%). Participants were 47% less likely to be working in the private sector (OR = 0.5.; 95% CI, 0.3–0.9, *p* < 0.018) than unemployed.

### 3.2. Demographic Characteristics and COVID-19 Vaccine Acceptance

In the bivariate analysis (see Table 3 below), acceptance of the COVID-19 vaccine seems to be slightly higher among males (Odds ratio, OR = 1.46; 95% CI, 1.03–2.06, *p* < 0.03) than among female participants. Acceptance was lower among those who had no formal education (OR = 2.25; 95% CI, 1.16–4.36, *p* < 0.02) compared with participants with a tertiary education. Older participants aged between 61 and 70 years (OR = 4.25; 95% CI, 1.42–6.82, *p* < 0.02) and above 70 years (OR = 4.15; 95% CI, 1.3–7.02, *p* < 0.02) had higher acceptance than participants aged between 18 and 30 years. Participants in rural areas were 29% less likely to be vaccinated than those in urban centers (OR = 0.71; 95% CI, 0.51–1.01, *p* < 0.02), while those who were working in the private sector were 41.9% less willing to be vaccinated than the unemployed (OR = 0.58; 95% CI, 0.2–0.86, *p* < 0.02). Participants who were single had increased vaccine acceptance compared with the married (OR: 4.03, 95% CI: 1.43–7.27, *p* < 0.001).

After adjustment in the multivariate analysis, vaccine acceptance was significantly associated with being male (AOR: 1.46, 95% CI: 1.24–1.59, *p* < 0.031), no formal education (AOR: 2.25, 95% CI: 1.15–4.39, *p* < 0.017), working in the private sector (AOR: 5.71, 95% CI: 0.19–10.88 *p* < 0.022), and income in the range of KES 0–999 (AOR: 2.345, 95% CI: 1.13–1.47, *p* < 0.02).

### 3.3. Health Belief Model and COVID-19 Vaccine Acceptance

Table 4 shows the scores of the four dimensions of the HBM. Compared with the participants who were not willing to be vaccinated, those who were willing to be vaccinated had significantly higher scores in perceived benefits (3.98 ± 0.61 vs. 2.82 ± 0.58, *p* < 0.001), perceived barriers (3.67 ± 0.56 vs. 2.82 ± 0.71), *p* < 0.002), and cues to action (3.79 ± 0.49 vs. 2.82 ± 0.71, *p* < 0.001). They had lower scores in perceived susceptibility (2.93 ± 0.65 vs. 3.96 ± 0.87, *p* < 0.001), and slightly lower scores in perceived severity (2.87 ± 0.75 vs. 3.24 (0.91) ± 0.91, *p* < 0.05).

### 3.4. Factors Influencing COVID-19 Vaccine Hesitancy

**Figure 1:**
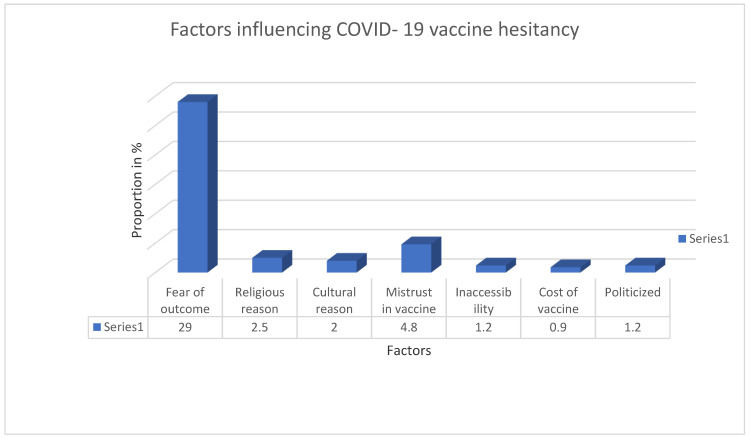
Factors influencing COVID-19 vaccine hesitancy.

The study showed that 336 (41.7%) of the participants were hesitant about receiving the COVID-19 vaccine. Of these, the majority (234, 69.6%) reported a fear of the outcome. This was followed by mistrust in the vaccine (11.6%), and religious reasons (6.0%). Other factors mentioned were inaccessibility and politicization of vaccines, each representing 3.0% of the participants (Figure 1).

### 3.5. Correlation Coefficients for Predictors of COVID-19 Vaccine Hesitancy

A bivariate Spearman correlation was run on the variables that pertained to vaccine hesitancy at a 95% confidence level (Table 5 below). It was established that hesitancy was correlated with fear of outcome of vaccine effects and was found to have strong positive correlation to not being vaccinated, (r = 0.89). In a similar manner, being afraid of contracting the COVID-19 had a negative correlation with vaccine hesitancy (r = 0.8), which implies that those who feel less afraid of contracting COVID-19 are less likely to get vaccinated and vice versa. Confidence in the efficacy and safety of the vaccine was significantly correlated with vaccine hesitancy (r = 0.74 and 0.71, respectively). This implies that the more people trust the safety and effectiveness of the vaccine, the more they are likely to get vaccinated, among other factors. Belief that healthcare workers have one’s best interest at heart was also found to have a positive correlation to vaccination (r = 0.082), implying that the more the trust in the intentions of healthcare workers, the higher the likelihood of getting vaccinated.

### 3.6. Strategies to Address COVID-19 Vaccine Hesitancy

Having identified the reasons for hesitancy and rejection of vaccination, strategies to address these were explored, with the following outcomes (Table 6 below).

A majority (63%) of the respondents supported the following strategies to improve vaccine acceptance: providing information in print, radio, television, and social media formats; providing toolkits, educational materials, and guidebooks to support community discussion about the COVID-19 vaccine; and making materials available in multiple languages. Sixty-one percent supported involving religious and traditional leaders, 59% supported health professionals to promote vaccination, and 56% supported building trust in the vaccines as strategies to improve uptake. Furthermore, 54% supported emphasizing the social benefits of vaccination as a strategy to improve vaccination. Fifty-two percent favored modularity and flexibility in the messaging on vaccination to allow customization by communities, 49% supported engaging opinion leaders such as celebrities, and 46% supported leveraging the role of health professionals to promote vaccination. Amplifying the endorsements of trusted community members to highlight the emerging norms that promote vaccination was supported by 45%.

## 4. Discussion

This study investigated correlates of vaccine acceptance and hesitancy in selected rural counties in western Kenya. Understanding correlates of vaccine acceptance and hesitancy is important as it could inform interventions to improve current and future pandemics and epidemics at various levels. Our study identified sociodemographic and psychosocial characteristics associated with hesitancy and acceptance of COVID-19 vaccines in rural populations in the western region of Kenya. The overall hesitancy rate was 41%. This was slightly higher than a previous multinational study which reported hesitancy rate of 37% [43]. Furthermore, the proportion of people willing to receive a COVID-19 vaccine in these rural counties was lower than the COVID-19 vaccine acceptance rates of 62% reported in South Asia (35), and 82% in the USA [41,45]. Another study conducted in 10 LMICs showed that more than 80% people in Asia, Africa, and South African regions expressed a willingness to accept a COVID19 vaccine [46]. Another study among refugees in Syria found that the COVID-19 vaccination rate was 50% in urban centers and above 90% in camps [38]. This variation could be attributed to people’s perceptions of risk of COVID-19 infection and severity, and the timing of the studies. Most of the previous surveys were conducted during the peak level of the pandemic and lockdown; the findings that more respondents were more willing to get vaccinated could be attributed to heightened fear of the unknown with a novel virus.

Considering sociodemographic characteristics, our study found that vaccine acceptance was significantly associated with being male, having no formal education, working in the private sector, and having an income of KES 0–999 (USD 0–9.51). Regarding the sex differentials, our results mirror a multinational survey which also reported lower odds of vaccine acceptance among females compared with males [43]. This observation could be because men are reported to have a higher risk of COVID-19 infection and because, culturally, women are perceived to be nurturers, making it more likely they seek medical help and value self-care. As such, they perceive themselves to be more susceptible and take measures to protect themselves.

Regarding education, the available literature reports contradictory findings, with some populations reporting higher odds of acceptance among participants with higher levels of education, while others linking higher education with lower levels of acceptance [10]. Our study was consistent with the latter finding. This paradox remains an ongoing discussion as the relationship between education and vaccine acceptance is complex across different contexts. Considering income, we found higher odds of acceptance among those in the lowest income bracket. This was in contrast to some studies in low- and middle-income countries which indicated higher acceptance associated with higher income [42].

Psychosocial factors including fear of side effects and mistrust have been reported to influence vaccine hesitancy across various populations [44,47]. Our study lends further credence to this established phenomenon by analyzing COVID-19 vaccine acceptance or hesitancy across the constructs of the Health Belief Model (HBM). Compared with the participants who are not willing to be vaccinated, those who are willing to be vaccinated had significantly higher scores in perceived benefits, perceived barriers, and cues to action. However, we also found that those willing to be vaccinated had relatively lower scores in perceived susceptibility and slightly lower scores in perceived severity. The findings from this study show that that perceived barriers, perceived benefits, and cues to action also had a direct impact on vaccination acceptance. The present study showed that the perceived benefits of COVID-19 vaccination were positively associated with COVID-19 vaccination acceptance, which is consistent with previous studies [48,49,50]. According to one study [44], the perceived benefits of COVID-19 vaccines can be disseminated through creative and impressive slogans in the media and in posters to highlight the significance of the COVID-19 vaccine in protecting health and in controlling the pandemic in the community. Perceived barriers were also significant determinants of the COVID-19 vaccination in Kenya. The identified barriers consisting of side effects, skepticism about the short vaccine development time, perceptions, and fears of the COVID vaccine as a ploy for population reduction, as well as concerns about the safety of the COVID-19 vaccine, have been reported in previous studies [51,52,53,54]. Consistent with a previous study [48], cues for action had a strong and positive effect on COVID-19 vaccination acceptance among community members in western Kenya.

Several findings in Asia have shown that the perceived risk or perceived susceptibility to an infection is associated with vaccine acceptance [50,51,52]. This study also indicated that those who had a higher perceived risk of being infected with COVID-19 were more likely to accept the vaccine. Other studies also reported that high perceived risk was associated with COVID-19 vaccine acceptance among general community members in Saudi Arabia [55] and in China [50]. Therefore, the perception of risk among communities in Kenya should be targeted since our study found that almost 64.5% of the respondents did not think they would be infected of COVID-19. Prominent among the drivers of hesitancy was a fear of vaccine side effects, which was reported by most of the respondents (69.6%). Further, our study found that vaccine hesitancy was significantly associated with fear of outcome, side effects and safety and the vaccine’s efficacy. These findings are comparable with those of previous reports [55,56,57,58]. Safety concerns about COVID-19 vaccination were a fundamental issue among 45% of respondents in the Indian population [58], 39.1% in Saudi Arabia [59], 47.8% in China, [50], and 46% in Qatar [60]. These findings could be attributed to the short period of development of the COVID-19 vaccines, which occasioned skepticism from the world’s anti-vaccination movements. Therefore, vaccine education campaigns with accurate transparent information on the efficacy and safety of the vaccines, among other things, should be communicated to the public in a timely manner to ensure all-inclusive immunization. However, with a virus like COVID-19, which was rapidly mutating and evolving, information changed quickly as more new things were learned about the virus and management of the pandemic, making information and risk communication quite a challenging task. This was costly in relation to building trust for scientific as well as professional information in the general population. Vaccine hesitancy continues to garner interest among researchers, policy makers, government leaders, and other stakeholders [61,62,63,64], especially now that the pandemic phase has passed and efforts to sustain prevention and deal with any new infection surges are likely to wane with time. Many lessons have been learned through this pandemic. Policymakers should also provide information and health education about vaccine safety to the public regularly to reduce public concerns about vaccine safety and effectively respond to future possible pandemics. On the other hand, religious beliefs and mistrust had insignificant effects on COVID-19 vaccine acceptance in the three counties in Kenya. Hence working with multi-sectoral stakeholders is paramount in dealing with such a broad-ranging pandemic.

## 5. Conclusions

The current results provide a view on the public responses to the COVID-19 vaccination in Kenya as vaccines were initially being rolled out. The current study suggests factors such as male gender, no formal education, working in the private sector, and low income (KES 0–999 (USD 0–9.6)) as significant variables that influence awareness of and possible acceptance of COVID-19 vaccination. Factors related to vaccine acceptance and hesitancy were of paramount importance as the pandemic surged, hence the need to address such issues and increase immunizations. The findings of this study may have implications for future strategies in promoting COVID-19 vaccine acceptance. The findings are helpful for stakeholders and strategic planners to intensify efforts to reduce vaccine hesitancy, improve health risk communication, increase and improve coordination of health education efforts in times of emergencies such as the COVID-19 pandemic, and improve uptake of vaccines for improved and better public health outcomes.

## Figures and Tables

**Table 1:** Sampling frame.

Kakamega	Kakamega Central	233
	Navakholo	199
Kisumu	Kisumu Central	138
	Nyando	129
Vihiga	Emuhaya	68
	Hamisi	90
	Total	857

**Table 2:** Demographic characteristics of the participants.

	Male	Female	n (%)	*p* Value	OR
Age					
18–30	156 (51.1)	149 (48.9)	305 (37.8)	0.78	0.8 (0.2–3.4)
31–40	162 (61.4)	102 (38.6)	264 (32.8)	0.023	0.5 (0.1–1.9)
41–50	50 (52.1)	46 (47.9)	96 (11.9)	0.659	0.7 (0.2–3.1)
51–60	39 (51.3)	37 (48.7)	76 (9.4)	0.63	0.7 (0.2–3.0)
61–70	28 (62.2)	17 (37.8)	45 (5.6)	0.35	0.5 (0.1–2.2)
Above 70	3 (27.3)	8 (72.7)	11 (1.4)	Ref	
**Education**					
None	31 (46.3)	36 (53.7)	67	0.098	1.7 (0.9–3.1)
Primary	53 (50)	53 (53)	106	0.163	1.5 (0.9–2.5)
Secondary	166 (49.6)	169 (50.4)	335	0.001	1.9 (1.3–2.7)
University	192	106	298	Ref	
**County**					
Kakamega	256 (60.4)	168 (39.6)	424	0.065	0.6 (0.4–1.0)
Kisumu	133 (48.7)	140 (51.3)	273	0.911	0.97 (0.6–1.6)
Vihiga	53 (48.6)	56 (51.4)	109	Ref	
**Employment**					
Civil servant	65 (57.5)	48 (42.5)	113	0.775	1.1 (0.7–1.7)
Working in private sector	64 (66.7)	32 (33.3)	96	0.018	0.5 (0.3–0.9)
Personal business	113 (59.5)	77 (40.5)	190	0.022	0.64 (0.4–0.9)
Unemployed	200 (49.1)	207 (50.9)	407	Ref	

OR—odds ratio, Ref—reference.

**Table 3:** Demographic characteristics and COVID-19 vaccine acceptance.

	COR	95% Cl	*p* Value	AOR	95% Cl	*p* Value
**Gender**						
Males	1.461	1.03–2.06	0.03	1.461	1.036–2.06	0.031
Female	Ref	Ref	Ref	Ref	Ref	Ref
**Age in years**						
18–30	3.497	0.38–32.27	0.27	3.497	0.412–29.69	0.251
31–40	4.401	0.48–40.25	0.19	4.401	0.521–37.146	0.173
41–50	3.378	0.36–32.05	0.29	3.378	0.385–29.637	0.272
51–60	2.551	0.26–25.08	0.42	2.551	0.284–22.936	0.403
61–70	4.246	0.42–42.42	0.02	4.246	0.455–39.616	0.204
Above 70	Ref	Ref	Ref	Ref	Ref	Ref
**Education**						
No education	2.253	1.16–4.36	0.02	2.253	1.155–4.398	0.017
Primary	1.682	0.94–3	0.08	1.682	0.933–3.032	0.084
Secondary	1.249	0.84–1.87	0.28	1.249	0.825–1.893	0.293
Tertiary education	Ref	Ref	Ref	Ref	Ref	Ref
**County**						
Kakamega	1.359	0.78–2.37	0.28	1.359	0.792–2.332	0.265
Kisumu	0.999	0.57–1.76	1	0.999	0.567–1.758	0.997
Vihiga	Ref	Ref	Ref	Ref	Ref	Ref
**Residence**						
Rural	0.714	0.51–1.01	0.04	0.714	0.502–1.017	0.062
Urban	Ref	Ref	Ref	Ref	Ref	Ref
**Occupation**						
Civil servant	0.59	0.28–1.24	0.16	0.59	0.28–1.243	0.166
Private sector	0.58	0.2–0.86	0.02	5.718	0.198–10.882	0.022
Self-employed	0.726	0.4–1.31	0.29	0.726	0.403–1.309	0.287
Unemployed	Ref	Ref	Ref	Ref	Ref	Ref
**Income** (KES/month)						
1–999	2.347	1.23–4.47	0.01	2.347	1.239–4.447	0.009
1000–9999	1.074	0.5–2.29	0.85	1.074	0.503–2.295	0.853
10,000–19,999	1.717	0.87–3.38	0.12	1.717	0.861–3.425	0.125
20,000–29,999	1.821	0.81–4.07	0.14	1.821	0.818–4.053	0.142
30,000–39,999	0.556	0.17–1.83	0.33	0.556	0.172–1.798	0.327
40,000–49,999	1.025	0.36–2.93	0.96	1.025	0.355–2.958	0.963
50,000–59,999	1.182	0.43–3.27	0.75	1.182	0.431–3.243	0.746
≥60,000	Ref	Ref	Ref	Ref	Ref	Ref
**Marital status**						
Single Married	4.025 Ref	1.43–7.27	0.001	4.75 Ref	0.431–9.43 Ref	0.001

COR—Crude odds ratio; AOR: the adjusted odds and odds ratios were derived from multivariate logistic regression models, CI: confidence interval. Exchange rate as at 23 August 2023: 1 USD = 144.75 KES (Kenyan Shilling).

**Table 4:** Correlates of COVID-19 vaccine acceptance and Health Belief Model (*n* = 806).

	Vaccination Acceptance		*p* Value	OR (95% CI)
	Yes	NO		
**Perceived susceptibility** **Perceived susceptibility score (Mean SD)**	2.93 (0.65)	3.96 (0.872)	0.001	
My chance of getting COVID-19 is high.	286 (35.5)	520 (64.5)	0.022	1.6 (0.23–2.97)
I believe there are other better ways to prevent disease than with vaccines.	380 (47.1)	426 (52.9)	0.082	1.18 (−0.59–0.23)
I believe that the use of alternative medicine eliminates the need for vaccination.	348 (43.1)	442 (56.9)	0.008	1.22 (−0.201–2.64)
**Perceived severity** **Perceived severity Score (Mean SD)**	2.87 (0.75)	3.24 (0.91)	0.04	
Do you know anyone who has had serious reaction to the vaccine?	251 (31.1)	553 (68.6)	0.002	0.69 (0.26–1.12)
Complications from COVID-19 are serious.	622 (77.1)	176 (22.9)	0.692	0.1 (−0.59–0.39)
I believe that catching COVID-19 does not cause severe health complications.	246 (30.5)	546 (69.5)	0.04	0.57 (−0.62–0.87)
I believe that catching COVID-19 does not prevent daily activities.	299 (37.1)	493 (61.2)	0.273	0.14 (−0.11,0.39)
I am concerned about death resulting from infection.	624 (77.4)	177 (22.0)	0.396	0.21 (−0.71,0.28)
**Perceived benefit ** **Perceived benefits score (Mean SD)**	3.98 (0.61)	2.82 (0.71)	0.01	
The benefits of COVID-19 vaccines are greater than the risks.	464 (57.6)	338 (43.4)	0.022	0.5 (−0.46–0.92)
Vaccination is a good idea because I feel less worried about catching COVID-19.	496 (61.5)	305 (38.5)	0.144	0.59 (−0.69–0.80)
Vaccination decreases my chances of getting COVID-19 or its complications.	524 (65.0)	281 (35)	0.003	0.69 (−1.14, −0.23)
**Perceived barriers score** **Perceived barriers (Mean SD)**	3.67 (0.56)	2.78 (0.6)	0.002	
I believe that COVID-19 vaccine causes side effects and would interfere with my usual activities.	519 (64.4)	285 (35.4)	0.089	0.39 (−0.06–0.84)
I am concerned about the efficacy of COVID-19 vaccination.	564 (70.0)	241 (30.0)	0.050	0.43 (−0.88–0.01)
Do you think COVID-19 is a biological weapon?	426 (52.9)	380 (47.1)	0.001	0.86 (0.45,1.29)
Unreliable due to short time of development.	557 (69.1)	248 (30.1)	0.039	0.21 (−0.25,0.67)
I am concerned about faulty/fake COVID-19 vaccines.	578 (71.70	228 (28.3)	0.329	0.23 (−0.24,0.70)
I prefer other ways of protection, e.g., herbal.	295 (36.6)	511 (63.4)	0.001	0.69 (0.27,1.12)
I am concerned that the COVID-19 pandemic is a conspiracy.	374 (46.4)	432 (53.6)	0.385	0.18 (−0.23,0.60)
I believe the vaccine jab is a population reduction strategy.	320 (39.7)	485	0.168	0.30 (−0.13,0.72)
**Cue to action** **Cues to action (Mean SD)**	3.79 (0.59)	2.28 (0.49)	0.001	
Have you heard about the COVID-19 vaccine?	706 (87.6)	100 (12.3)	0.296	0.29 (−0.84–0.26)
Do you think that the COVID-19 vaccine is needed?	600 (74.4)	205 (25.6)	0.044	0.44 (−0.92–0.04)
Do you feel you know which vaccines you should get yourself?	270 (33.5)	536 (66.5)	0.232	0.26 (−0.69,0.17)
I feel confident that I know enough to guide my decision about getting COVID-19 vaccine.	534 (66.3)	272 (33.7)	0.589	−0.12 (−0.56–0.32)
Do you trust your health care provider to honestly tell you about the risks and benefits of vaccines?	554 (68.7)	252 (32.3)	0.046	0.70 (0.21–1.90)
Do you trust the vaccine advice your main health care provider gives you?	535 (66.6)	271 (33.4)	0.02	0.79 (0.28–0.21)
Do you think you can protect people you know and love by getting COVID-19 vaccine?	562 (69.7)	244 (30.3)	0.1	−0.38 (−0.84–0.07)
	**Vaccination Acceptance**		***p* Value**	**OR (95% CI)**
	**Yes**	**NO**		
**Perceived susceptibility** **Perceived susceptibility score (Mean SD)**	2.93 (0.65)	3.96 (0.872)	0.001	
My chance of getting COVID-19 is high.	286 (35.5)	520 (64.5)	0.022	1.6 (0.228–2.97)
I believe that there are other better ways to prevent disease than with vaccines.	380 (47.1)	426 (52.9)	0.082	1.18 (−0.595–0.228)
I believe that the use of alternative medicine eliminates the need for vaccination.	348 (43.1)	442 (56.9)	0.008	1.22 (−0.201–2.635)
**Perceived severity** **Perceived severity Score (Mean SD)**	2.87 (0.75)	3.24 (0.91)	0.04	
Do you know anyone who has had a serious reaction to the vaccine	251 (31.1)	553 (68.6)	0.002	0.69 (0.255–1.12)
Complications from COVID-19 are serious.	622 (77.1)	176 (22.9)	0.692	0.1 (−0.586–0.389)
I believe that catching COVID-19 does not cause severe health complications.	246 (30.5)	546 (69.5)	0.04	0.57 (−0.618–0.873)
I believe that catching COVID-19 does not prevent daily activities.	299 (37.1)	493 (61.2)	0.273	0.14 (−0.109, 0.386)
Death resulting from infection	624 (77.4)	177 (22.0)	0.396	0.21 (−0.708,0.28)
**Perceived benefit ** **Perceived benefits score (Mean SD)**	3.98 (0.61)	2.82 (0.71)	0.01	
The benefits of COVID-19 vaccines are greater than the risks.	464 (57.6)	338 (43.4)	0.022	0.5 (−0.459–0.915)
Vaccination is a good idea because I feel less worried about catching COVID-19.	496 (61.5)	305 (38.5)	0.144	0.59 (−0.698–0.802)
Vaccination decreases my chances of getting COVID-19 or its complications.	524 (65.0)	281 (35)	0.003	0.69 (−1.137, 0.233)
**Perceived barriers score** **Perceived barriers (Mean SD)**	3.67 (0.56)	2.78 (0.6)	0.002	
I believe that the COVID-19 vaccine causes side effects and would interfere with my usual activities.	519 (64.4)	285 (35.4)	0.089	0.39 (−0.06–0.842)
I am concerned about the efficacy of COVID-19 vaccination.	564 (70.0)	241 (30.0)	0.050	0.43 (−0.875–0.012)
Do you think COVID-19 is a biological weapon?	426 (52.9)	380 (47.1)	0.001	0.86 (0.426, 1.289)
It is unreliable due to the short time of development.	557 (69.1)	248 (30.1)	0.039	0.21 (−0.254, 0.667
I am concerned about faulty/fake COVID-19 vaccines.	578 (71.70	228 (28.3)	0.329	0.23 (−0.235, 0.701)
I prefer other ways of protection, e.g., herbal.	295 (36.6)	511 (63.4)	0.001	0.69 (0.271, 1.115)
I am concerned that the COVID-19 pandemic is a conspiracy.	374 (46.4)	432 (53.6)	0.385	0.18 (−0.23, 0.597)
I believe the vaccine jab is a population reduction strategy.	320 (39.7)	485	0.168	0.3 (−0.125, 0.719)
**Cue to action** **Cues to action (Mean SD)**	3.79 (0.59)	2.28 (0.49)	0.001	
Have you heard about the COVID-19 vaccine?	706 (87.6)	100 (12.3)	0.296	0.29 (−0.839–0.255)
Do you think that COVID-19 vaccine is needed?	600 (74.4)	205 (25.6)	0.044	0.44 (−0.917–0.042)
Do you feel you know which vaccines you should get yourself?	270 (33.5)	536 (66.5)	0.232	0.26 (−0.688, 0.166)
I feel confident that I know enough to guide my decision about getting COVID-19 vaccine.	534 (66.3)	272 (33.7)	0.589	−0.12 (−0.555–0.315)
Do you trust your health care provider to honestly tell you about the risks and benefits of vaccines?	554 (68.7)	252 (32.3)	0.046	0.7 (0.21–1.9)
Do you trust the vaccine advice your main health care provider gives you?	535 (66.6)	271 (33.4)	0.02	0.79 (0.28–0.213)
Do you think you can protect people you know and love by getting COVID-19 vaccine?	562 (69.7)	244 (30.3)	0.1	−0.38 (−0.836–0.073)

OR—odds ratio; The variables with *p*-values of more than 0.05 in the univariate analysis were not included in the multivariate logistic regression models.

**Table 5:** Correlation coefficients for hesitancy.

Factors	Coefficient (r)	*p*-Value
Fear of the outcome	0.89	0.012
Religious reason	0.076 *	0.032
Mistrust of vaccine	0.071 *	0.048
Complications from COVID-19 are serious.	−0.72 *	0.042
I am afraid of getting COVID-19.	0.80 *	0.026
I am concerned about the efficacy of COVID-19 vaccination.	0.74 *	0.036
I am concerned about the safety of COVID-19 vaccination.	0.71 *	0.045
Politicized	0.02	0.116
I am concerned that the COVID-19 pandemic is a conspiracy.	−0.083	0.019
I believe the vaccine jab is a population reduction strategy.	0.085	0.079
Do you believe your health care provider has you and your children’s best interest at heart?	−0.082 *	0.021

* Significant at 0.05 level.

**Table 6:** Strategies to address COVID-19 vaccine hesitancy and improve acceptance.

Strategy	Yes	No	Total
Making social norms in favor of vaccination more salient	258 (32.00)	548 (68.00)	806
Highlighting new and emerging norms in favor of vaccination	361 (44.79)	445 (55.21)	806
Leveraging the role of health professionals	374 (46.40)	432 (53.60)	806
Supporting health professionals to promote vaccination	478 (59.31)	328 (40.70)	806
Amplifying endorsements from trusted community members	366 (45.40)	440 (54.60)	806
Involving religious and traditional leaders	492 (61.04)	314 (38.96)	806
Consistent, flexible and modular messaging to allow local authorities to tailor it to specific communities	417 (51.74)	389 (48.26)	806
Building timely trust in vaccines	452 (56.08)	354 (43.92)	806
Leveraging anticipated regret in communications	251 (31.14)	555 (68.86)	806
Emphasizing the social benefits of vaccination	434 (53.85)	372 (46.15)	806
Engage thought and opinion leaders, such as celebrities, to help promote COVID-19 vaccination acceptance and uptake	398 (49.38)	408 (50.62)	806
Messaging in various languages and graphical elements to increase motivation, counter misinformation, and overcome barriers to vaccination	463 (57.44)	343 (42.56)	806
Include all media formats and educational materials to support community discussions on vaccination	514 (63.77)	292 (36.23)	806
Other	153 (18.98)	652 (80.89)	806

## Data Availability

The datasets used and/or analyzed during the current study are contained within this article. Any additional data or clarification available upon reasonable request.
